# Three-Dimensional Morphometric Trajectories Following Lip Lift With or Without Fat Grafting in Facial Feminization Patients

**DOI:** 10.1093/asjof/ojag135

**Published:** 2026-07-01

**Authors:** Victoria Kong, Andrew Salib, Martin Kauke-Navarro, Emily Parker, Jake Moscarelli, Omar Allam, Samira Glaeser-Khan, Samuel Knoedler, Michael Alperovich

## Abstract

**Background:**

Subnasal lip lifts address perioral aging, but long-term quantitative data on postoperative changes and permanence remain limited.

**Objectives:**

The aim of this study was to provide the first 3-dimensional (3D) evaluation of morphometric changes after subnasal lip lift ± autologous fat grafting over time.

**Methods:**

A retrospective cohort (2022-2025) of facial feminization patients undergoing subnasal lip lift ± fat grafting was analyzed. Standardized VECTRA 3D imaging measured philtral height, vermilion height and width, nasal base width, columella–labial angle, surface area, and lip volume preoperatively and at serial postoperative intervals. Early changes (3 months) were compared using *t* tests and longitudinal trajectories with linear mixed-effects models.

**Results:**

Forty patients were included (lip lift alone, *n* = 10; lip lift + fat grafting, *n* = 30). Both cohorts showed significant early philtral shortening (−2.92 ± 1.53 mm, *P* = .013 vs −5.95 ± 1.77 mm, *P* < .001) and increased upper vermilion surface area (1.40 ± 0.86 cm^2^, *P* = .022 vs 1.42 ± 1.23 cm^2^, *P* = .022). Fat grafting produced greater early volumetric augmentation (1.18 ± 0.86 vs 0.53 ± 0.25 cc), although between-group differences did not reach statistical significance. Mixed-effects modeling showed significant divergence in permanence. Philtral shortening showed 43% retention by 24 months (*P* = .003), whereas volumetric and surface area gains approached zero by 22 and 24 months, respectively.

**Conclusions:**

There is measurable philtral shortening after subnasal lip lift. However, partial relengthening occurs over time. Additionally, early gains in vermilion display and lip volume decrease substantially by 2 years. Fat grafting enhances early augmentation but shows limited long-term retention.

**Level of Evidence: 3 (Therapeutic):**

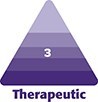

As patients age, the upper lip and philtrum undergo cutaneous elongation, volume depletion, thinning, and soft-tissue descent.^1,2^ Surgical lip lifting is widely used to address these changes and restore youthful characteristics by shortening the philtrum and increasing upper vermilion show.^3,4^ The most common approach is the subnasal technique with a “bull's horn” excision pattern, with autologous fat grafting frequently performed concurrently to augment perioral volume.^5^ Because demand for these procedures increases, understanding the temporal behavior and long-term stability of these aesthetic changes is increasingly important for operative planning and patient counseling.^1^

Despite the popularity of lip lifting, long-term quantitative data on postoperative durability remain limited. Existing literature is primarily descriptive, cadaveric, or based on 2-dimensional photography, and most studies report outcomes at a single time point.^4,6,7^ Furthermore, no previous work has provided an objective, temporal evaluation of changes in lip dimensions following lip lift with adjunctive fat grafting. Although surgeons frequently observe the attenuation of their early postoperative results, the magnitude and rate of this regression have not been quantitatively characterized.

Three-dimensional (3D) surface imaging offers a precise and reproducible method for measuring linear, surface area, and volumetric changes. The objective of this study was to use 3D morphometric analysis to quantify postoperative changes after subnasal lip lift with or without fat grafting and to characterize the durability and trajectory of these changes over time, including their direction, rate, and permanence. To our knowledge, this is the first study to model postoperative trajectories following lip lift using serial 3D imaging as well as the first to evaluate soft-tissue behavior following combined lip lift and autologous fat grafting. By defining the extent and rate of postoperative attenuation, this work aims to provide surgeons with evidence that can help inform patient counseling, procedural planning, and long-term aesthetic management.

## METHODS

### Patient Population

A retrospective cohort study was performed of patients who underwent facial feminization surgery, including a subnasal lip lift with or without autologous fat grafting between March 2022 and June 2025, by a single surgeon at a large academic institution. Facial feminization surgery is a group of surgical procedures primarily performed for transgender women to transform masculine facial features into feminine features, which most commonly includes forehead contouring and orbital contouring.^8,9^ Inclusion criteria were age ≥18 years, with standardized preoperative and at least 1 postoperative 3D photogrammetric image (VECTRA, Canfield Scientific, Parsippany, NJ). Patients were excluded if they underwent any facial procedures between postoperative imaging sessions, including injectable filler or second-stage feminization, that could confound morphometric measurements.

Postoperative imaging was obtained at multiple time points, with each patient contributing between 1 and 4 time points (range, 1-27 months) of postoperative imaging. All patients provided informed consent under a protocol approved by the Yale University IRB (HIC# 2000031685).

### Surgical Technique

All procedures were performed under general endotracheal anesthesia with the patient placed in a supine position. A modified subnasal bullhorn approach was used, with 3 to 8 mm of cutaneous resection, depending on preoperative philtral height and the desired degree of lip eversion. Symmetric markings were drawn alar-to-alar, tracing along the nasal sill and columellar base. After injection of local anesthesia, cutaneous and subcutaneous tissue were excised to the level of the orbicularis oris. A myocutaneous flap was advanced to achieve philtral shortening.

When performed, autologous fat grafting was harvested from either the abdomen or thighs, processed by Telfa (Covidien, Dublin, Ireland) rolling, and injected into the lips using standard Coleman cannulas with a microdroplet technique. Fat was deposited primarily into the vermilion, with supplemental injection at the white roll in select cases. The cutaneous lip was not grafted. Because of the retrospective nature of the study, consistent documentation of intraoperative graft volume, injection plane, and the precise distribution between vermilion-only vs combined vermilion and white roll injection was not available. These variables could not be correlated with postoperative volumetric outcomes.

Postoperatively, patients were advised to maintain head elevation for 1 week. Patients were also prescribed a 7-day course of a first-generation cephalosporin. Nonabsorbable sutures were removed between postoperative days 5 and 7. Dietary restrictions included a liquid diet for the first 24 h, followed by a transition to a full liquid and subsequent soft diet.

### 3D Photogrammetric Assessment

Standardized 3D images were taken under uniform lighting and positioning. The quantitative morphometric analysis was conducted through the Canfield VECTRA Analysis Module. The VECTRA system has been previously validated for facial morphometry, demonstrating a high degree of repeatability, reproducibility, and submillimeter accuracy, with a root mean square error of 0.43 mm for linear measurements.^10^ The imaging system contributes nonsignificantly to overall measurement variance (3.11%) and demonstrates high reliability in manual landmarking (up to 98.7%).^11,12^ Therefore, the small magnitude changes observed in this study exceed the VECTRA system's technical error threshold.

**Figure 1.**
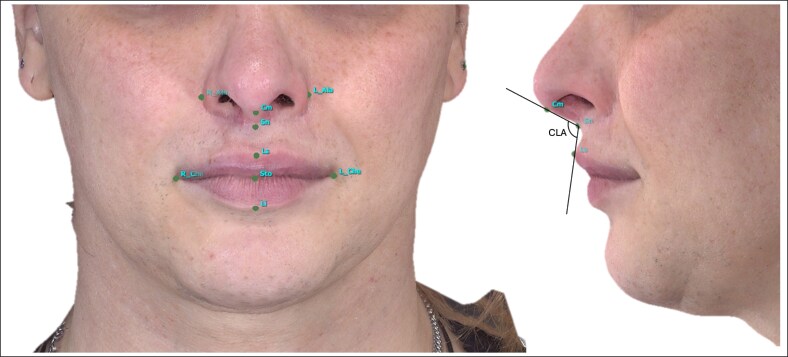
Anatomical landmarks used for morphometric analysis in a representative patient (Patient A, a 38-year-old transgender female) who underwent facial feminization surgery, including subnasal lip lift with autologous fat grafting. Photograph taken 11 months postoperatively. CLA, columella–labial angle (measurement); Cm, columella; L_Ala, left ala; L_Che, left chelion; Li, labrale inferius; Ls, labrale superius; R_Ala, right ala; R_Che, right cheilion; Sn, subnasale; Sto, stomion.

On each image, anatomical landmarks, including the bilateral alares, subnasale, labrale superius, stomion, labrale inferius, bilateral chelion, and columella, were identified manually (Figure 1).

Linear measurements (philtral height, upper vermilion height, lower vermilion height, vermilion width, and nasal base width) were calculated as straight-line distances between defined landmarks. The columella–labial angle (CLA) was also calculated from defined landmarks. All measurement definitions are summarized in Table 1. To quantify the long-term durability of the procedure, the proportion of surgical resection preserved at each postoperative time point (*t*) was calculated using Equation 1:


(1) $$\begin{array}{c} \mathrm{Resection}\;\mathrm{preservatio}n_{t}\;=\;\left(\frac{H_{\mathrm{Pre}}-\;H_{t}}{H_{\mathrm{Resected}}}\right)\times 100\text{\%}, \end{array}$$

where *H*_Pre_ and *H_t_* represent the preoperative and postoperative philtral heights measured on 3D imaging, and *H*_Resected_ represents the vertical dimension of the cutaneous excision measured intraoperatively. Vermilion surface area (upper, lower, and total) was calculated following the methodology described by Rho et al, using triangulated surface mapping based on manually traced vermilion borders.^13^

**Table 1.** Anatomical Definitions of Outcome Variables

Outcome variable	Definition
Philtrum height	Linear distance from subnasale (Sn) → labrale superius (Ls)
Upper vermilion height	Linear distance from Ls → stomion (Sto)
Lower vermilion height	Linear distance from Sto → labrale inferius (Li)
Vermilion width	Linear transverse distance between right and left cheilion (R_Che → L_Che)
Nasal base width	Linear distance between right and left alar-facial grooves (R_Ala → L_Ala)
Columella–labial angle	Angle formed by columella (Cm) → Sn and Sn → Ls in the midsagittal plane
Surface area: total, upper, lower lips	Automated measurement of the vermilion area traced along vermilion borders using VECTRA software loop function (cm^2^)
Volume: total, upper, lower lips	Absolute volume difference between postoperative surface relative to baseline within vermilion borders using aligned 3-dimensional surfaces (cc)

**Figure 2.**
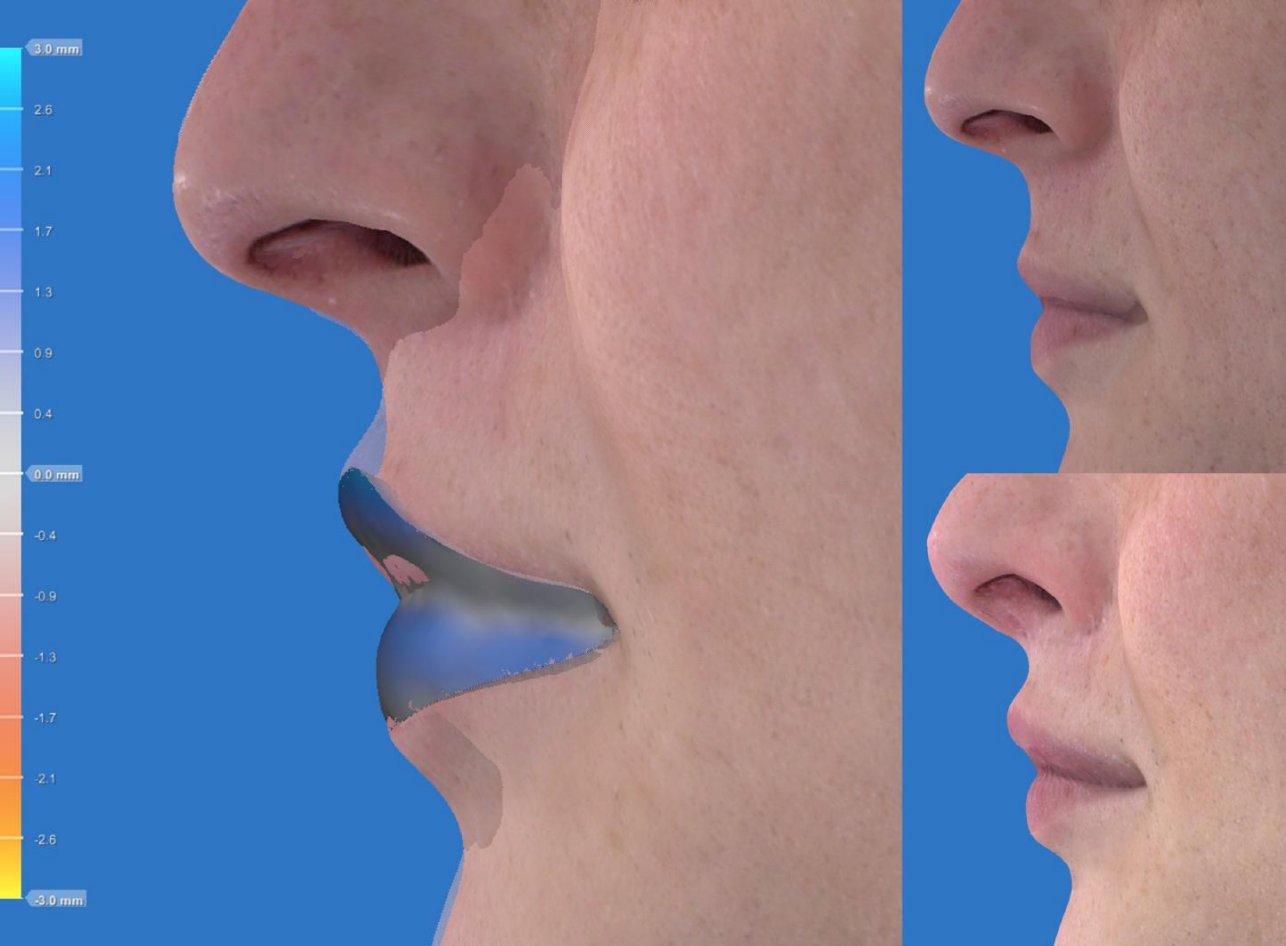
Three-dimensional (3D) volumetric comparison of preoperative and postoperative lip surfaces in a 38-year-old transgender female (Patient A) presenting for facial feminization, 11 months following subnasal lip lift with autologous fat grafting. (A) Semitransparent overlay of the postoperative surface onto the preoperative surface after surface registration, with the region of volumetric difference within the vermilion border rendered opaquely to depict postoperative augmentation. Color mapping indicates magnitude of projection change, with increasing intensity corresponding to greater postoperative projection (up to 3 mm). (B) Preoperative 3D surface. (C) Postoperative 3D surface following subnasal lip lift with autologous fat grafting.

Lip volumetric change was calculated by overlaying each postoperative 3D surface at each follow-up with its corresponding preoperative surface. Mesh alignment was accomplished by either manually selecting surgically unaltered facial regions (eg, forehead, nose, chin, or ears) or by landmark-based alignment followed by automated full surface matching registration. Absolute volume differences within the vermilion border were computed to determine upper, lower, and total lip volume changes at each postoperative interval. An example of this process is shown in Figure 2.

### Statistical Analysis

Data analysis was performed using R version 4.5.1 (R Core Team, 2025). Descriptive statistics were used to summarize patient demographics and changes in outcome variables. Continuous variables were compared using independent samples *t* tests and categorical variables using Fisher's exact test. Paired *t* tests assessed early postoperative changes within each group, whereas between-group comparisons of change were assessed using independent *t* tests.

Early stable postoperative analyses included only patients whose postoperative scan occurred at 3 months. This interval was selected to assess morphometric changes after resolution of early postoperative edema and stable fat graft integration. Patients without imaging in this interval were excluded from this subanalysis but were retained for the overall longitudinal mixed-effects modeling. Immediate postoperative data (0-3 months) are provided for reference in Supplemental Table 1.

Because each patient contributed multiple postoperative scans (each between 1 and 4 time points) at nonuniform intervals, postoperative trajectories were examined using linear mixed-effects models with subject-level random intercepts to account for within-patient correlation and baseline variability. This approach allowed for the calculation of change (slope, *β*) for each morphometric variable, providing a mathematical description of the direction and permanence of postoperative outcomes. Postoperative time (in months) was modeled as a continuous variable. Procedure type (lip lift alone vs lip lift and fat grafting) was evaluated as both a main effect and an interaction with time. Fat grafting was not found to be a significant determinant of longitudinal outcomes (all *P* > .05). As such, final trajectory models were fit using pooled data from both the lip lift–only and lip lift and fat grafting cohort. Similarly, rhinoplasty was not a significant predictor of nasal base width and was therefore not retained as a covariate. Alternative nonlinear and spline-based models were explored using Bayesian Information Criterion (BIC) and likelihood ratio tests, shown in Supplemental Table 2. *P*-values for model comparisons were adjusted using the Benjamini–Hochberg procedure to control for multiple comparisons. These comparisons confirmed that linear trajectories provided the most parsimonious fit for the observed postoperative soft-tissue behavior. Although lower vermilion height showed nominal improvement with nonlinear modeling, all outcomes yielded a ΔBIC < 6, indicating insufficient evidence to favor more complex models.^14^ Accordingly, linear mixed-effects models were retained.

Average values for each outcome variable at 12 and 24 months were derived from the modeled postoperative trajectories generated from longitudinal mixed-effects analyses, accompanied by corresponding 95% CIs. These values represent model-based estimates derived from all available longitudinal data rather than observations restricted to patients imaged exactly at those time points. The fixed effect of time was used to test the significance of temporal trends. A *P*-value of <.05 was considered statistically significant.

## RESULTS

### Patient and Perioperative Characteristics

Forty patients met inclusion criteria, of whom 10 (25%) underwent lip lift alone and 30 (75%) underwent lip lift with concomitant autologous fat grafting. All participants (*n* = 40; 100%) were transgender females. The mean age was 39.0 ± 11.7 years (range, 20-74 years) and the mean BMI was 25.4 ± 4.0 kg/m^2^. Racial composition was 67.5% White (*n* = 27), 12.5% Black/African American (*n* = 5), and 20% identifying as other (*n* = 8). Ten percent of patients identified as Hispanic. Baseline demographic and clinical characteristics were similar between groups (Table 2).

**Table 2.** Baseline Patient Characteristics by Procedure Type

	Lip lift only	Lip lift + Fat grafting	*P*-value	Total
	*n* = 10	*n* = 30		*n* = 40
Age, years (mean ± SD)	44.8 ± 15.0	37.1 ± 10.0	.156	39.0 ± 11.7
Gender			1.0	
Male	0 (0)	0 (0)		0 (0)
Female	10 (100)	30 (100)		40 (100)
BMI, kg/m^2^ (mean ± SD)	24.4 ± 3.9	25.8 ± 4.1	.371	25.4 ± 4.1
Race			1.0	
White	7 (70.0)	20 (66.7)		27 (67.5)
Black	1 (10.0)	4 (13.3)		5 (12.5)
Other	2 (20.0)	6 (20.0)		8 (20.0)
Hispanic	0 (0)	4 (13.3)	.556	4 (10)
Comorbidities				
Smoker	0 (0)	0 (0)	1.0	0 (0)
Diabetes mellitus	1 (10.0)	1 (3.3)	.589	2 (5.0)

Values are given as *n* (%), unless otherwise indicated. SD, standard deviation.

The mean preoperative philtral height was 17.9 ± 3.0 mm and decreased to 13.8 ± 1.7 mm at 3 months postoperatively. The mean philtral resection measured 6.4 ± 1.1 mm overall. The difference between the excision length and the net clinical shortening likely reflects tissue relaxation and relapse observed within this postoperative interval. There were no significant differences in resection between cohorts, with 6.9 ± 0.8 mm resected for lip lift alone and 6.3 ± 1.2 mm for lip lift with fat grafting (*P* = .093). The mean postoperative follow-up was 6.7 ± 5.2 months (range, 1-27 months).

### Early Stable Postoperative Morphometric Change

Twleve patients had available imaging data at the 3-month time point (lip lift alone, *n* = 5; lip lift with fat grafting, *n* = 7). The analysis of these early postoperative images provided exploratory insights into early morphometric trends following the resolution of surgical edema (Table 3). Both cohorts demonstrated the expected pattern of philtral shortening and increased upper vermilion display. The magnitude of philtral height reduction was nearly twice as large in the lip lift and fat-grafted cohort, at −5.95 ± 1.77 mm compared with −2.92 ± 1.53 mm in the lip lift–only cohort (*P* = .011). Similarly, gains in vermilion height were more pronounced with fat grafting, with a nearly 2-fold increase in the upper lip (3.26 ± 2.24 vs 1.90 ± 1.81 mm, *P* = .27) and a substantial increase in the lower lip (1.63 ± 1.21 vs 0.23 ± 1.26 mm, *P* = .088). Early changes in transverse and angular metrics were minimal.

**Table 3.** Early Stable Postoperative Morphometric Changes (3 Months Postoperative)

Outcome variable	Lip lift only		Lip lift + fat grafting		Between group *P*-value^a^
	*n* = 5		*n* = 7		
	Mean ± SD Δ	*P-*value	Mean ± SD Δ	*P-*value	
Philtrum height (mm)	-2.92 ± 1.53*	.013*	-5.95 ± 1.77*	<.001*	.011*
Upper vermilion height (mm)	1.90 ± 1.81	.079	3.26 ± 2.24*	.008*	.27
Lower vermilion height (mm)	0.23 ± 1.26	.708	1.63 ± 1.21*	.012*	.088
Vermilion width (mm)	0.98 ± 1.59	.24	0.70 ± 2.67	.516	.823
Nasal base width (mm)	1.04 ± 0.47*	.008*	−0.66 ± 1.96	.407	.063
Columella–labial angle (°)	2.22 ± 11.85	.732	−7.73 ± 10.13	.09	.212
Vermilion surface area (cm^2^)					
Total	2.17 ± 1.66*	.043*	2.32 ± 1.62*	.009*	.882
Upper	1.40 ± 0.86*	.022*	1.42 ± 1.23*	.022*	.966
Lower	0.77 ± 1.06	.177	0.89 ± 1.08	.071	.851
Vermilion volume (cc)					
Total	0.53 ± 0.25*	.004*	1.18 ± 0.86*	.006*	.103
Upper	0.33 ± 0.10*	.001*	0.52 ± 0.43*	.009*	.292
Lower	0.21 ± 0.19*	.035*	0.65 ± 0.48*	.005*	.052

SD, standard deviation.

^a^Between-group comparisons are exploratory given limited sample size.

^*^Statistical significance, *P* < .05.

Surface area increased comparably in both cohorts, most notably in the upper lip. Total vermilion surface area expanded by 2.17 ± 1.66 cm^2^ after lip lift alone, compared with 2.32 ± 1.62 cm^2^ with fat grafting (*P* = .882). Upper lip surface area similarly increased in both cohorts (1.40 ± 0.86 vs 1.42 ± 1.23 cm^2^, *P* = .966), whereas lower lip surface area changes were more modest (0.77 ± 1.06 vs 0.89 ± 1.08 cm^2^, *P* = .851).

Early postoperative increases in measured lip volume were observed in both groups (all *P* ≤ .035), with consistently greater increases in patients who underwent concomitant fat grafting. The addition of autologous fat grafting was associated with more than double the total lip volume gain (1.18 ± 0.86 cc) compared with lip lift alone (0.53 ± 0.25 cc; *P* = .103). In the lip lift–only cohort, this likely reflects volumization from tissue redistribution rather than additive volume augmentation. This trend was most prominent in the lower lip, where the observed volume gain in the lip lift and fat grafting cohort was triple that of the lip lift–only cohort (0.65 ± 0.48 vs 0.21 ± 0.19 cc, *P* = .052), approaching statistical significance. Of note, comparison of volumetric gains in the immediate postoperative period (0-3 months; see Supplemental Table 1) suggests that these gains may be partially attenuated by 3 months, consistent with the expected resolution of postoperative edema and early fat remodeling.

Given the limited sample size at this interval, these early between-group comparisons are primarily descriptive. Together, these data suggest that lip lift alone produces measurable early surface area and volume enhancement, but the addition of autologous fat grafting appears to offer a greater magnitude of early volumetric and vertical gains.

### Longitudinal Trends and Durability of Aesthetic Change

**Figure 3.**
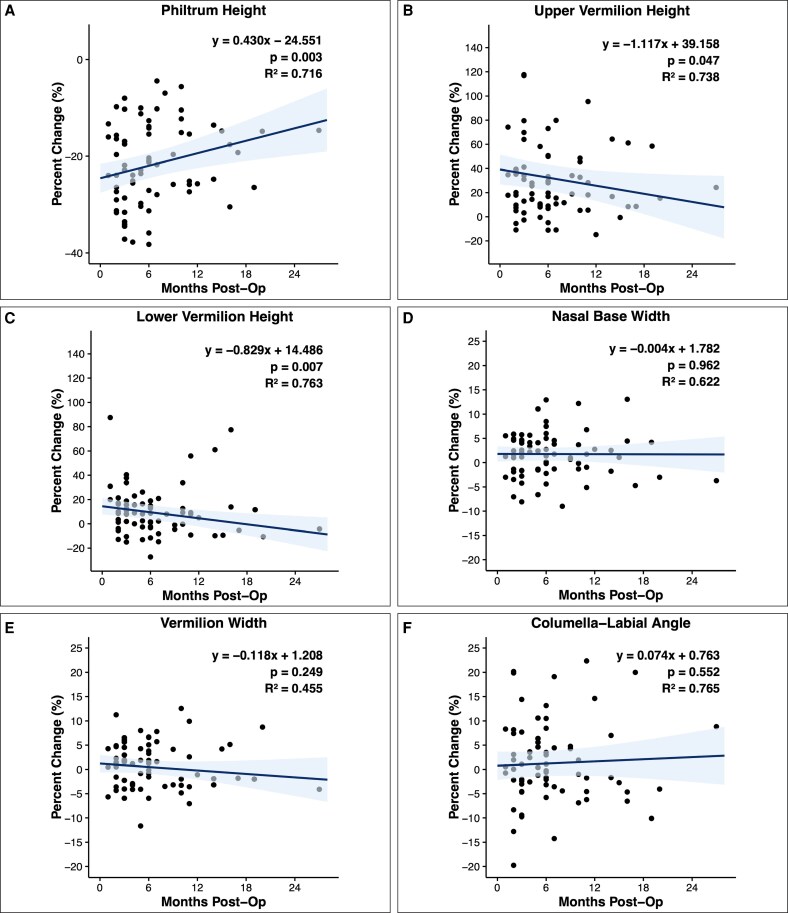
Linear mixed-effects models demonstrating longitudinal percent change from baseline for each morphometric variable following subnasal lip lift with or without fat grafting. Models incorporate subject-level random intercepts; shaded areas represent 95% CIs. Follow-up intervals reflect a total cohort of 40 patients, with data points available for 33 patients at 0 to 6 months, 24 at 6 to 12 months, 7 at 12 to 18 months, and 3 at >18 months. (A) Philtrum height. (B) Upper vermilion height. (C) Lower vermilion height. (D) Nasal base width. (E) Vermilion width. (F) Columella–labial angle. Percent change (%) relative to baseline is plotted against postoperative time in months.

**Figure 4.**
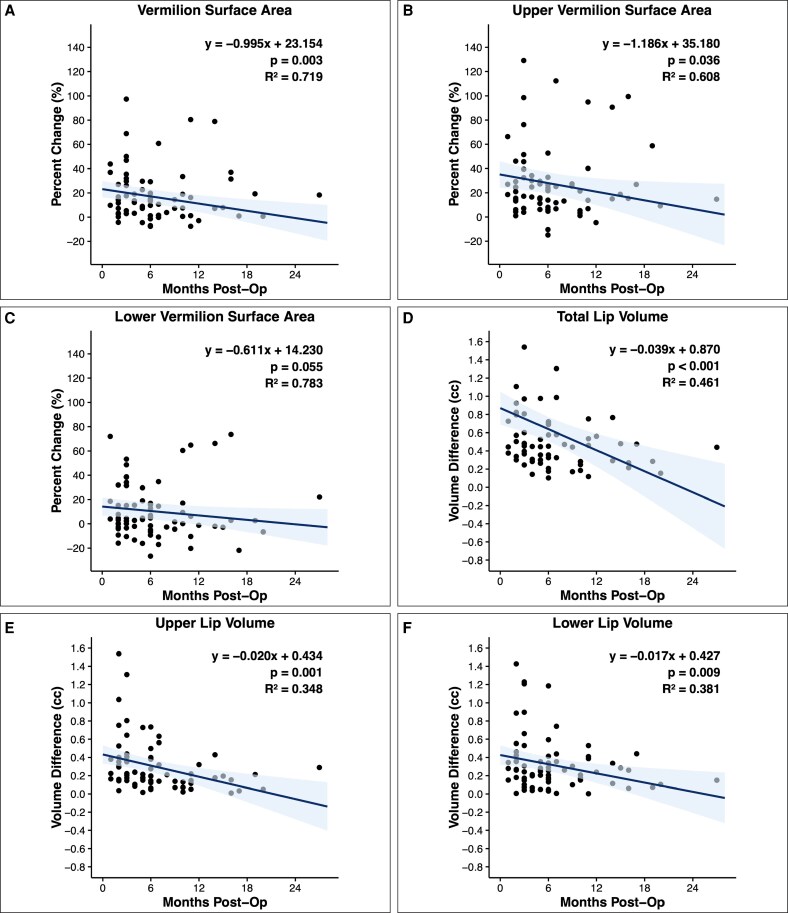
Linear mixed-effects models demonstrating longitudinal percent change from baseline for each morphometric variable following subnasal lip lift with or without fat grafting. Models incorporate subject-level random intercepts; shaded areas represent 95% CIs. Follow-up intervals reflect a total cohort of 40 patients, with data points available for 33 patients at 0 to 6 months, 24 at 6 to 12 months, 7 at 12 to 18 months, and 3 at >18 months. (A) Total vermilion surface area. (B) Upper vermilion surface area. (C) Lower vermilion surface area. (D) Total lip volume. (E) Upper lip volume. (F) Lower lip volume. Percent change (%) relative to baseline is plotted against postoperative time in months.

Mixed-effects modeling showed that fat grafting did not alter long-term trajectories of all outcome variables (all *P* > .05); hence, lip lift–only and lip lift and fat grafting cohorts were pooled for longitudinal models. Because postoperative imaging occurred at heterogeneous intervals (1-27 months), the reported 12- and 24-month values represent model-predicted estimates derived from the fitted longitudinal trajectories of all patients’ combined data (Table 4). This approach allowed estimation of standardized postoperative landmarks irrespective of individual follow-up timing. Data point availability across follow-up windows was as follows: 33 of 40 patients (82.5%) had data in the 0- to 6-month window, 24 (60%) in the 6- to 12-month window, 7 (17.5%) in the 12- to 18-month window, and 3 (7.5%) beyond 18 months. Across all evaluated variables, the models demonstrated a consistent pattern of loss of early postoperative gains. Representative regression plots with linear fits, model equations, and CIs for all outcome variables further illustrates these rates and directions of change over time (Figures 3, 4).

**Table 4.** Predicted Changes in Perioral Metrics at 12 and 24 Months Based on Longitudinal Modeling

	Predicted change (95% CI)		
Outcome variable (% change unless noted^a^)	12 months	24 months	*P*-value^b^
Philtrum resection retained (%)	56.31 (45.09, 67.53)*	42.52 (25.06, 59.98)*	.007*
Philtrum height	−19.39 (−22.47, −16.31)*	−14.23 (−19.79, −8.67)*	.003*
Upper vermilion height	25.75 (13.09, 38.42)*	12.34 (−9.86, 34.54)*	.045*
Lower vermilion height	4.54 (−2.41, 11.49)*	−5.40 (−17.34, 6.54)*	.007*
Nasal base width	1.74 (0.09, 3.39)	1.69 (−1.45, 4.83)	.962
Vermilion width	−0.21 (−2.06, 1.63)	−1.63 (−5.49, 2.22)	.249
Columella–labial angle	1.65 (−1.37, 4.66)	2.53 (−2.59, 7.65)	.552
Vermilion surface area			
Total	11.21 (4.19, 18.24)*	−0.73 (−13.36, 11.91)*	.003*
Upper	20.94 (9.87, 32.02)*	6.71 (−14.80, 28.21)*	.036*
Lower	6.89 (−0.85, 14.64)	−0.44 (−13.39, 12.50)	.055
Vermilion volume difference (cc)			
Total	0.41 (0.23, 0.59)*	−0.06 (−0.45, 0.34)*	.001*
Upper	0.19 (0.09, 0.29)*	−0.06 (−0.28, 0.16)*	.001*
Lower	0.23 (0.12, 0.33)*	0.02 (−0.21, 0.26)*	.009*

^a^All outcomes are expressed as percent change from baseline unless otherwise specified. Philtrum resection retained is reported as absolute percentage (%), and vermilion volume difference as absolute change (cc).

^b^ *P*-values reflect the overall effect of time (baseline → 12 months → 24 months) in the longitudinal model.

^*^Statistical significance, *P* < .05.

### Philtrum Height

**Figure 5.**
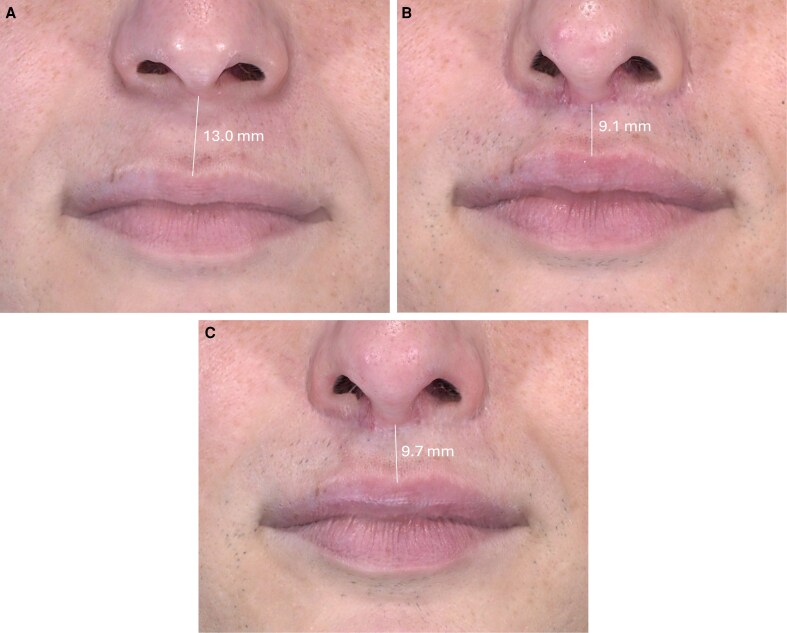
Progressive increase in philtral length following lip lift (7 mm skin excision) of Patient A, a 38-year-old transgender female presenting for facial feminization. (A) Preoperative appearance. (B) Five months postoperatively. (C) At 11 months, early elongation of the philtrum and soft-tissue settling are noted.

Although philtral height remained reduced postoperatively at all intervals compared with preoperative measurements, the magnitude of reduction significantly decreased over time. Philtral height gradually, but significantly, increased at a gradual rate of ∼*β* = +.43%/month (*P* = .003, Figure 3A). Model-derived reduction was −19.39% at 12 months (95% CI, −22.47 to −16.31) and −14.23% at 24 months (95% CI, −19.79 to −8.67). Similarly, the proportion of surgical resection preserved (calculated as Eq. 1) declined from 56.31% at 12 months to 42.52% at 24 months (*P* = .0074). Figure 5 illustrates a representative clinical example.

### Vermilion Height

Upper vermilion height demonstrated an early postoperative increase, followed by a gradual decline (*β* = −1.12%/month, *P* = .047; Figure 3B). Predicted augmentation from mixed-effects modeling decreased from +25.75% at 12 months (95% CI, 13.09-38.42) to +12.34% at 24 months (95% CI, −9.86 to 34.54). Lower vermilion height exhibited a significant downward trend as well (*β* = −.83%/month, *P* = .007; Figure 3C), shifting from +4.54% at 12 months (95% CI, −2.41 to 11.49) to −5.40% at 24 months (95% CI, −17.34 to 6.54).

### Nasal Base Width, Vermilion Width, and CLA

Nasal base width, vermilion width, and CLA exhibited longitudinal stability (*P* = .962, *P* = .249, and *P* = .552, respectively). This suggests that the procedures have minimal impact on nasal–labial angular relationships as well as on transverse lip morphology and nasal base width over the study interval.

### Surface Area

Total vermilion surface area also demonstrated a significant decline over time (*β* = −1.0%/month, *P* = .003, Figure 4A). Mixed-effects modeling estimated that total surface area remained significantly greater than baseline at 12 months (+11.21%, 95% CI, 4.19-18.24) but regressed toward baseline by 24 months (−0.73%, 95% CI, −13.36 to 11.91). The upper lip area exhibited the greatest early relative expansion at 12 months (+20.94%, 95% CI, 9.87-32.02) with partial regression by 24 months (+6.71%, 95% CI, −14.80 to 28.21). The rate of surface area decrease for the upper lip was ∼*β* = −1.2%/month (*P* = .036; Figure 4B). The lower lip area trended similarly (*β* = −.6%/month; Figure 4C) but was borderline statistically significant (*P* = .055).

### Volume

Postoperative total lip volume similarly remained significantly greater than baseline at the model-predicted 12 month interval (total +0.41 cc, upper +0.19 cc, and lower +0.23 cc) but significantly regressed over time (total *β* = −.04 cc/month, *P* < .001; upper *β* = −.02 cc/month, *P* = .001; lower *β* = −.02 cc/month, *P* = .009; see Figure 4D-F). By 24 months, all volumes approached baseline, with estimated volume differences relative to baseline of −0.06 cc (95% CI, −0.45 to 0.34) for total lips, −0.06 cc (95% CI, −0.28 to 0.16) for upper lips, and +0.02 cc (95% CI, −0.21 to 0.26) for lower lips.

### Complications

There were no major complications, including fat necrosis, infection, seroma, hematoma, fat embolism, prolonged paresthesia, alar flaring, or nasal sill widening. Three patients (7.5%) experienced adverse scarring at the subnasal incision. This was addressed with intralesional triamcinolone (Kenalog, Bristol-Myers Squibb) injections, resulting in satisfactory scar maturation. No other complications were noted.

## DISCUSSION

To our knowledge, we present the first study to model longitudinal postoperative soft-tissue trajectories following subnasal lip lift, with or without autologous fat grafting, using serial 3D photogrammetry.^4,6,7,15^ Our principal finding is that philtral height remains shortened relative to baseline at all postoperative intervals, although partial regression was observed. Although the vertical shortening of the philtrum appears relatively durable, gains in vermilion display and fullness eventually returned to near-preoperative levels by 24 and 22 months, respectively. Given that these gains regress to baseline by 2 years, the long-term utility of autologous fat grafting in the lips must be carefully considered. Clinicians should counsel patients that the long-term stability of fat grafting in this highly mobile region may be limited. By quantifying the rate, direction, and permanence of perioral metrics, this work provides a predictive mathematical framework for what has previously been qualitatively observed but unmeasured.

Early postoperative changes in philtral height and vermilion display align with previous studies that utilized 2-dimensional (2D) photographs. Nagy et al described ∼62% to 71% retention of philtral shortening at 24 months in a cohort of 52 patients.^6^ Marechek et al demonstrated a 69% improvement in vermilion-to-philtrum ratio at a single postoperative time point around 14 months (*n* = 55).^7^ Of note, our study is unique in its departure from 2D photographic-based methodology utilized by previous authors. Unlike 2D methods, 3D photogrammetry is capable of quantifying depth and curvature changes. This leads to superior reliability in locating anatomical landmarks and the ability to quantify volumetric loss. Through the utilization of 3D photogrammetry and mixed-effects modeling, we provide a more granular characterization of postoperative outcomes, quantifying both the rate and durability of change. Our data demonstrate that 56% of philtral resection was retained at 12 months and 43% at 24 months. Philtral height remained significantly shorter than baseline throughout the study period.

The relapse pattern observed of both increased philtral height and diminished vermilion display likely reflects a combination of biologic and mechanical forces, including scar maturation, attenuation of initial dermal fixation, and the natural elongation associated with aging.^6,16^ Furthermore, the surgical technique utilized in this cohort involved myocutaneous flap advancement without muscle resection, which can lead to “bunching” of the orbicularis oris at the nasal base. This accumulated tissue bulk, combined with the dynamic pull of the muscle in use, likely creates a mechanical vector that contributes to philtral lengthening over time. Alternative techniques such as the T-shaped muscle resection described by Pan or horizontal muscle strip removal noted by Santanchè and Bonarrigo address this bulk through direct excision and extensive undermining toward the Cupid's bow.^17,18^ These maneuvers facilitate tension-free redraping by shifting the structural load to deep muscular suspension, which may mitigate the relapse in philtral height observed with simple flap advancement.

The clinical significance of these millimeter-level shifts in philtral and vermilion height is supported by the work of Kempa et al, who utilized eye-tracking and surveys to identify the most attractive and feminine upper lip proportion as a 1:2 ratio of upper vermilion height to philtral height.^19^ Significant differences in observer attention were noted for ratios rated less attractive, even for small deviations such as 1:1.6. In this study, the small quantitative changes of longitudinal lengthening of the philtrum and concomitant decrease in vermilion height may reach the threshold of perceptibility, potentially contributing to the diminished perceived attractiveness of the perioral region.

In light of these findings, the role of adjunctive treatments to preserve philtral shortening may be considered. Although the effect of onabotulinumtoxinA (BTX-A) on philtral height has not yet been addressed in the literature specifically, its use to weaken the orbicularis oris is well-established in aesthetic practice.^20,21^ Furthermore, BTX-A injections into the orbicularis oris during cleft lip repair have been shown to produce narrower scars by reducing tension during the healing phase.^22^ We hypothesize that postoperative BTX-A could similarly immobilize the musculature, weakening the pull that contributes to philtral lengthening. However, this remains speculative and warrants prospective study.

Interestingly, we observed small but statistically significant early increases in measured lip volume even in the lip lift–only cohort, averaging ∼0.5 cc. Although this represents a measured volumetric increase as determined by 3D surface overlay, no volume was added in this group. This finding suggests that after vertical advancement of the upper lip, redistribution of tension across the orbicularis oris may lead to measurable volumization of the lip independent of fat grafting.^16,23^ The horizontal tensile load may be lesser in magnitude. Cadaveric work by Patel et al showed stepwise gains in vermilion area with increasing lift magnitude (*n* = 13), and our clinical 3D measurements corroborate these mechanical relationships in vivo.^4^ However, our longitudinal modeling indicates that these early volumetric gains regress. We observed a return to baseline levels by 24 months for the overall cohort. From a mechanistic standpoint, this pattern likely reflects resolution of postoperative edema and gradual soft-tissue remodeling.

Although small, the clinical relevance of these submilliliter volumetric shifts is supported by recent findings by Othman et al, who demonstrated that subtle lip volumization (with typical injection volumes of 0.5 to 2 mL in a standard filler session) is associated with significantly higher observer ratings across multiple interpersonal domains, including youthfulness, attractiveness, and confidence.^24-26^ Even the 0.5 cc volume gain from lip lift alone may possibly provide a perceptible improvement to how patients are perceived by casual observers. Autologous fat grafting demonstrated a significant early additive effect by nearly doubling early total lip volume compared with lip lift alone. However, based on our model estimates, this augmented volume progressively diminished and was no longer appreciable by ∼22 months postoperatively. Previous studies by Tuin et al and Schipper et al utilizing 3D photogrammetry to assess longitudinal volume changes following fat grafting similarly found favorable short-term augmentations in the lips, although their estimates showed faster resorption with a return to preoperative volumes within a year.^15,27^ Their studies were also limited by sample size, with *n* = 12 and *n* = 1 for the lips, respectively. Taken together, the present study suggests that lip fat grafting serves as a temporary contour adjunct, not a durable volumizing strategy. The lips are highly mobile, with ongoing mechanical shear within the tissue, and contain relatively little native fat.^28^ These factors may contribute to accelerated graft resorption rather than sustained adipose retention.

Although fat grafting itself did not alter long-term trajectories of outcome variables in the mixed-effects models, this finding should be interpreted with caution. Limited statistical power, combined with inherent heterogeneity in fat graft volume and injection technique, may have contributed to the absence of an observed difference. These findings do not necessarily imply therapeutic equivalence, and larger, prospective studies are warranted.

Transverse metrics including nasal base width, vermilion width, and CLA remained stable over time. Rho et al similarly demonstrated variable CLA change posthyaluronic filler injection over 3 months.^13^ Of note, our results stand in contrast to Marechek et al, who observed a statistically significant 2.2% increase in nasal base width following subnasal lip lift.^7^ Nasal base widening is a common preoperative concern, but our study presents data that may be of reassurance.^7^ In the context of careful flap design and alar base management, our data suggest that clinically meaningful nasal widening does not appear to be an expected consequence of the subnasal lip lift.

Strengths of our study include the use of standardized serial 3D imaging across multiple postoperative time points, as well as mixed-effect modeling analysis that accounts for patient-level variability and irregular follow-up intervals. We provide a comprehensive characterization of change across linear, surface, and volumetric domains. However, our study has several limitations. First, the modest overall sample size and cohort imbalance, particularly in the early postoperative subanalysis, reduce power and require that between-group differences be interpreted cautiously. These subanalyses should be viewed as exploratory. Therefore, the absence of evidence for a long-term benefit of fat grafting in this cohort should not be misconstrued as definitive proof of equivalence. Second, the reported 12- and 24- month values are model-derived projections based on linear mixed-effects modeling. Although this approach allows estimation of standardized postoperative measurements from heterogeneous follow-up data, it is subject to modeling assumptions and potential errors arising from data inputs, particularly given the limited number of patients with long-term follow-up beyond 18 months. Third, the magnitude of many observed differences is small (eg, 0.5-1 cc, millimeter-level changes). Although these findings were statistically significant and align with existing literature suggesting that changes at this scale may be visually perceptible, the absence of patient-reported outcome measures (PROMs) fails to confirm this perceptibility within this specific cohort.^19,24^ Fourth, the single-surgeon design as well as selection from a facial feminization patient cohort may limit external generalizability. Facial feminization patients present with greater facial skeletal dimensions, thicker soft tissue, and the influence of concurrent procedures.^29-31^ Imaging intervals at the patient level were heterogenous, although this was adjusted for through the mixed models. Fifth, because of the retrospective design, we were unable to correlate volumetric outcomes with intraoperative fat graft volumes or injection plane. Future prospective studies with larger sample sizes are warranted to integrate PROMs, correlate intraoperative fat graft volumes and injection planes, and include lateral alar-to-vermilion vertical heights for more granular assessments of lateral lip morphology, long-term stability, and overall patient perception and satisfaction.

## CONCLUSIONS

Philtral shortening after subnasal lip lift is durable after partial regression, but most early gains in vermilion height, surface area, and lip volume approach baseline by 2 years. Although fat grafting offers greater early augmentation, it is not a permanent volumetric solution in the mobile perioral region. By characterizing the trajectory of these changes, this study offers practical evidence for surgical planning and managing expectations for patients undergoing aesthetic surgery.

## Supplementary Material

ojag135_Supplementary_Data
