# Supplementary material for: Three-Dimensional Morphometric Trajectories Following Lip Lift With or Without Fat Grafting in Facial Feminization Patients
Source: Aesthet Surg J Open Forum. 2026 Jul 1;8:ojag135. doi: 10.1093/asjof/ojag135 (PMC13361976; doi:10.1093/asjof/ojag135)
Supplement: ojag135_Supplementary_Data [file ojag135_supplementary_data.zip › Supplementary Table 1.docx]

**Supplementary Table 1.** Immediate Postoperative Morphometric Changes (0–3 Months Postoperative)

| Outcome Variable | Lip Lift Only | | Lip Lift + Fat Grafting | | Between Group  p-value |
| --- | --- | --- | --- | --- | --- |
|  | N = 5 | | N = 20 | |  |
|  | Mean ± SD Δ | p value | Mean ± SD Δ | p value |  |
| Philtrum Height (mm) | **–3.09 ± 1.34** | **0.007** | **–4.59 ± 1.78** | **<0.001** | 0.072 |
| Upper Vermilion Height (mm) | **2.23 ± 1.54** | **0.032** | **2.22 ± 1.92** | **<0.001** | 0.990 |
| Lower Vermilion Height (mm) | 0.43 ± 1.42 | 0.540 | **1.07 ± 1.21** | **<0.001** | 0.388 |
| Vermilion Width (mm) | 0.82 ± 1.62 | 0.318 | 0.66 ± 2.40 | 0.234 | 0.859 |
| Nasal Base Width (mm) | **1.25 ± 0.61** | **0.010** | –0.07 ± 1.64 | 0.858 | **0.010** |
| Columella-Labial Angle (°) | 1.61 ± 10.35 | 0.745 | –1.40 ± 12.07 | 0.610 | 0.592 |
| Vermilion Surface Area (cm^2^) |  |  |  |  |  |
| Total | **2.27 ± 1.52** | **0.029** | **1.47 ± 1.33** | **<0.001** | 0.330 |
| Upper | **1.46 ± 0.76** | **0.013** | **1.01 ± 0.88** | **<0.001** | 0.290 |
| Lower | 0.80 ± 1.03 | 0.155 | **0.46 ± 0.86** | **0.028** | 0.518 |
| Vermilion Volume (cc) |  |  |  |  |  |
| Total | **0.53 ± 0.25** | **0.005** | **1.01 ± 0.72** | **<0.001** | **0.013** |
| Upper | **0.33 ± 0.10** | **0.001** | **0.49 ± 0.41** | **<0.001** | 0.069 |
| Lower | **0.20 ± 0.19** | **0.037** | **0.52 ± 0.40** | **<0.001** | **0.011** |

Bold values indicate statistical significance (p < 0.05).
