# Supplementary material for: Three-Dimensional Morphometric Trajectories Following Lip Lift With or Without Fat Grafting in Facial Feminization Patients
Source: Aesthet Surg J Open Forum. 2026 Jul 1;8:ojag135. doi: 10.1093/asjof/ojag135 (PMC13361976; doi:10.1093/asjof/ojag135)
Supplement: ojag135_Supplementary_Data [file ojag135_supplementary_data.zip › Supplementary Table 2.docx]

**Supplementary Table 2.** Model Fit Comparison: Linear, Polynomial, and Spline Specifications

|  | BIC | | | p_adj_​ | p_adj​_ |
| --- | --- | --- | --- | --- | --- |
| Variable | Linear | Poly | Spline | (Lin vs. Poly) | (Lin vs. Spline) |
| Philtrum Height (mm) | 515.2 | 519.4 | 523.3 | 0.859 | 0.875 |
| Upper Vermilion Height (mm) | 719.5 | 723.1 | 726.0 | 0.511 | 0.532 |
| Lower Vermilion Height (mm) | 630.5 | 626.6 | 625.8 | **0.039** | **0.012** |
| Vermilion Width (mm) | 450.3 | 454.6 | 458.6 | 0.879 | 0.875 |
| Nasal Base Width (mm) | 427.6 | 430.3 | 434.5 | 0.312 | 0.533 |
| Columella-Labial Angle (°) | 493.9 | 494.2 | 499.3 | 0.137 | 0.443 |
| Total Vermilion Surface Area (cm^2^) | 635.3 | 633.9 | 636.9 | 0.078 | 0.137 |
| Upper Vermilion Surface Area (cm^2^) | 707.0 | 709.4 | 712.1 | 0.306 | 0.443 |
| Lower Vermilion Surface Area (cm^2^) | 644.2 | 646.1 | 650.0 | 0.282 | 0.443 |
| Total Lip Volume (cc) | 114.2 | 116.6 | 120.5 | 0.249 | 0.468 |
| Upper Lip Volume (cc) | 29.7 | 30.0 | 34.1 | 0.142 | 0.373 |
| Lower Lip Volume (cc) | 37.3 | 41.4 | 45.4 | 0.672 | 0.803 |

Bold values indicate statistical significance (p < 0.05).

Model fit was compared using Bayesian Information Criterion (BIC) and likelihood ratio tests (LRTs). Lower BIC values indicate improved balance between goodness-of-fit and model parsimony. P_adj_ values were calculated using the Benjamini-Hochberg procedure to adjust for multiple comparisons.
